# The association between smoking and unfavorable outcomes in acute ischemic stroke patients with mechanical thrombectomy

**DOI:** 10.18332/tid/119229

**Published:** 2020-04-06

**Authors:** Zhihong Zhao, Zheng Zhao, Xiaohan Zheng, Xiang Li, Xuemei Li, Chaoping Huang, Yajie Shan, Linda Nyame, Mako Ibrahim, Xiaoping Gao, Hui Liang, Jue Hu, JianJun Zou

**Affiliations:** 1 Department of Neurology, The First Affiliated Hospital (People’s Hospital of Hunan Province), Hunan Normal University, Changsha, China; 2 Department of Pharmacy, Nanjing First Hospital, Nanjing Medical University, Nanjing, China; 3 School of Basic Medicine and Clinical Pharmacy, China Pharmaceutical University, Nanjing, China; 4 Department of Neurology, Changsha Central Hospital, Changsha, China

**Keywords:** smoking, stroke, mechanical thrombectomy, intracranial hemorrhages, cardioembolism

## Abstract

**INTRODUCTION:**

Little is known about the relationship between smoking and clinical outcomes in acute ischemic stroke (AIS) patients undergoing mechanical thrombectomy (MT). The outcomes could depend on different stroke subtypes. The aim of this study was to investigate whether smoking affected differently the outcomes in patients with different stroke subtypes who received MT.

**METHODS:**

AIS patients who underwent MT were prospectively enrolled from three hospitals between January 2014 and December 2018. Smokers were defined as current users of cigarettes. The stroke subtypes were classified according to TOAST criteria. Outcome measurements included treatment effects, intracerebral hemorrhage (ICH), and functional outcomes at 3 months. The effects of smoking on outcomes were assessed by logistic regression analysis.

**RESULTS:**

A total of 128 AIS patients with MT were enrolled, including 64 smokers and 64 non-smokers. Logistic regression analysis indicated that smoking was related to higher risk of In-hospital ICH (OR=4.31; 95% CI: 1.10–16.96; p=0.036) in patients with cardioembolism subtype. Furthermore, smoking was also associated with lower rates of mild stroke at discharge (OR=0.07; 95% CI: 0.02–0.31; p<0.001) and functional independence (OR=0.13; 95% CI: 0.03–0.56; p=0.006) in patients with cardioembolism subtype.

**CONCLUSIONS:**

In AIS patients undergoing MT, smoking could be related to a higher risk of In-hospital ICH and lower rates of mild stroke at discharge and functional independence if their stroke subtype is cardioembolism.

## INTRODUCTION

Smoking is a well-known independent and modifiable risk factor for the development of acute ischemic stroke (AIS)^1-5^. Although hazardous effects are found on cerebrovascular health and cerebral infarction^6^, recently many studies have reported that smokers had better clinical outcomes and lower risk of intracranial hemorrhage (ICH) than non-smokers in AIS patients treated with intravenous thrombolysis (IVT)^7-11^, described as ‘Smoker’s paradox’. Most research has shown that the paradox of smoking can be explained by differences in demographic and clinical characteristics between smokers and non-smokers, such as younger age, ethnicity, etiological factors and lower frequency of cardiovascular risk factors^12-14^. However, little is known about the relationship between smoking and clinical outcomes after mechanical thrombectomy (MT), which is recommended by the current clinical practice guideline for the treatment of intracranial large-artery occlusive strokes^15-18^. Therefore, the association between smoking and MT still needs to be clarified.

We assume that the smoking–MT relationship could be associated with differences in demographic and clinical characteristics at baseline (e.g. subtypes of stroke) in AIS patients. We therefore conducted a multicenter prospective study with in-depth analysis of clinical data to examine the relationship between smoking and clinical outcomes of ischemic stroke after MT in overall Chinese AIS patients, including large artery atherosclerosis and cardioembolism stroke patients.

## METHODS

### Study population

Acute ischemic stroke patients who underwent MT were consecutively enrolled at Nanjing First Hospital, Hunan Provincial People’s Hospital, and Changsha Central Hospital, between January 2014 and December 2018. The study was performed according to the principles of the Helsinki Declaration 1975 and was approved by the Ethics Committee in Nanjing First Hospital, Hunan Provincial People’s Hospital, and Changsha Central Hospital. Written informed consent was obtained before enrollment.

Eligible patients for MT adhered to the following criteria: 1) age ≥18 years; 2) had a primary diagnosis of AIS; 3) onset of neurological symptoms <6 hours; 4) intracranial large artery occlusion evaluated by magnetic resonance angiography; and 5) National Institutes of Health Stroke Scale (NIHSS) score ≥5 on admission. Exclusion criteria were set as follows: 1) history of intracranial hemorrhage within 3 weeks; 2) any active bleeding or bleeding diathesis; 3) blood glucose concentration <2.8 or >22.0 mmol/L; 4) platelet count <10000/μL; and 5) severe hepatic or renal dysfunction. Smokers were defined as current users of cigarettes. Baseline characteristics were collected, including age, gender, tirofiban treatment, diabetes mellitus, previous cardiovascular disease, NIHSS score on admission, onset to treatment time (OTT), fasting blood glucose, platelet count, stroke locations, and stroke subtypes. The stroke subtypes were classified according to the Trial of ORG 10172 in Acute Stroke Treatment (TOAST) criteria: large artery atherosclerosis (LAA), small artery occlusion (SAO), cardioembolism (CE), and stroke of other determined or undetermined cause. All patients underwent brain imaging by magnetic resonance imaging (MRI) or computer tomography (CT) scan. Stroke subtypes classification was based on the brain imaging and clinical features and was performed by at least two experienced neurologists. A total of 250 AIS patients who underwent MT were initially recruited and then propensity score matching analysis was applied to the correction for disbalance in baseline characteristics. Briefly, smokers were matched to non-smokers by the nearest neighbor matching without replacement at 1:1 fixed ratio and the caliper value was set at 0.12. Eventually, a total of 128 patients were enrolled in the subsequent statistically analyses. Because of very few patients with stroke of determined cause, they were combined with those in the stroke of undetermined cause subtype and named together as the ‘Other’ group. Furthermore, there were no patients in the stroke of small artery occlusion subtype, and therefore all patients in this study were classified into three stroke subtypes (LAA, CE, and Other).

### Outcome measurements

*Treatment effects:* NIHSS scores at 24 hours, at 3 days, and at discharge. Mild stroke was defined^19^ as having a score of 0–4.

*Intracerebral hemorrhage:* 1) symptomatic intracranial hemorrhage (sICH) was evaluated according to ECASS2 criteria; 2) any In-hospital ICH, evaluated by computed tomography scan post MT or any time if neurologic deterioration occurred.

*Functional outcomes:* A modified Rankin Scale (mRS) score at 3 months was obtained by telephone follow-up and performed by well-trained and certified assessors. Minimal symptom and functional independence were defined as having a mRS score of 0–1 and 0–2, respectively.

### Statistical analyses

Baseline characteristics were compared between smokers and non-smokers in all patients with MT. For continuous variables, mean ± SD (or medians with interquartile range for skewed distribution) was used to summarize data, and two-tailed t-test or Mann-Whitney U test was performed to detect differences between groups. For categorical variables, frequencies and percentage were used to summarize data, and inter-group comparisons were performed by Pearson’s chi-squared test or Fisher’s exact test, where appropriate. The effects of smoking status on treatment effects, intracerebral hemorrhage and functional outcomes were assessed by logistic regression analysis. A p-value <0.05 was considered as indicating a significant difference. All statistical analyses were conducted using SPSS Version 23.0 (IBM Corp).

## RESULTS

### Baseline characteristics

A total of 128 acute ischemic stroke patients with MT were eligible for this study. Among them, 64 (50.0%) were current smokers. Based on their stroke subtype, 75 (58.6%) patients were in the LAA subgroup, 48 (37.5%) patients were in CE subgroup, and 5 (3.9%) patients were in the ‘Other’ subgroup. Baseline characteristics of smokers and non-smokers in all patients and in each ischemic stroke subtype are summarized in Table 1. Because of only 5 patients in the ‘Other’ subtype, inter-group comparisons could not be performed and therefore this subtype was ruled out in subsequent logistic regression analysis. In the LAA subgroup, the age of stroke occurrence was significantly younger in the smoking group than in the non-smoking group (63 vs 68 years, p<0.047). Apart from this, all baseline characteristics had no statistical difference between smokers and non-smokers either in all patients or in each stroke subtype (p>0.05).

**Table 1:** Baseline characteristics in smoking versus non-smoking patients with different ischemic stroke subtypes, Nanjing, China, 2014–2018 (N=128)

*Characteristics*	*All Patients (n=128)*			*LAA (n=75)*		

	*Smokers (n=64)*	*Non-smokers (n=64)*	*p*	*Smokers (n=40)*	*Non-smokers (n=35)*	*p*
Age (years)	65.2±9.6	66.9±15.8	0.472	63.0±8.4	68.0±12.4	0.047
Male gender	63 (98.4)	63 (98.4)	1.000	40 (100)	34 (97.1)	0.467
Treatment with tirofiban	22 (34.4)	18 (28.1)	0.446	17 (42.5)	10 (28.6)	0.210
Diabetes mellitus	8 (12.5)	14 (21.9)	0.160	7 (17.5)	8 (22.9)	0.563
Arterial hypertension	45 (70.3)	43 (67.2)	0.703	31 (77.5)	25 (71.4)	0.546
Hypercholesterolemia	5 (7.8)	4 (6.3)	1.000	4 (10.0)	2 (5.7)	0.679
Atrial fibrillation	18 (28.1)	20 (31.3)	0.699	1 (2.5)	2 (5.7)	0.596
Coronary heart disease	13 (20.3)	19 (29.7)	0.221	4 (10.0)	8 (22.9)	0.130
Previous TIA/stroke	1 (1.6)	1 (1.6)	1.000	0 (0)	0 (0)	-
Previous cerebral infarction	12 (18.8)	8 (12.5)	0.330	6 (15.0)	6 (17.1)	0.801
Previous cerebral hemorrhage	3 (4.7)	1 (1.6)	0.619	3 (7.5)	0 (0)	0.243
NIHSS on admission	13 (7–20)	14 (10–19)	0.372	10 (6–18)	14 (9–20)	0.154
OTT, min	330 (244–510)	322 (216–387)	0.158	394 (263–531)	350 (250–500)	0.500
Fasting blood glucose, mmol/L	6.11 (5.49–7.27)	6.30 (5.23–7.19)	0.941	6.17 (5.51–7.11)	6.43 (5.32–8.96)	0.435
Platelets (×1000/μL)	182 (149–221)	189 (145–223)	0.918	197 (169–236)	192 (146–225)	0.254
PT/INR	1.00 (0.95–1.09)	1.00 (0.93–1.12)	0.867	0.97 (0.91–1.04)	0.99 (0.93–1.06)	0.285
Intravenous thrombolysis	28 (43.8)	33 (51.6)	0.376	16 (40.0)	19 (54.3)	0.216
Anterior circulation stroke	45 (70.3)	45 (70.3)	1.000	22 (55.0)	22 (62.9)	0.491
Posterior circulation stroke	19 (29.7)	19 (29.7)	1.000	18 (45.0)	13 (37.1)	0.491
Stroke etiology (TOAST)						
LAA	40 (62.5)	35 (54.7)	0.592			
CE	21 (32.8)	27 (42.2)				
Other	3 (4.7)	2 (3.1)				
Age (years)	69.6±10.0	68.9±15.0	0.858	-	-	-
Male gender	20 (95.2)	27 (100)	0.438	3 (100)	2 (100)	-
Treatment with tirofiban	4 (19.0)	7 (25.9)	0.733	1 (33.3)	1 (50.0)	1.000
Diabetes mellitus	1 (4.8)	6 (22.2)	0.118	0 (0)	0 (0)	-
Arterial hypertension	13 (61.9)	18 (66.7)	0.732	1 (33.3)	0 (0)	1.000
Hypercholesterolemia	1 (4.8)	1 (3.7)	1.000	0 (0)	1 (50.0)	0.400
Atrial fibrillation	16 (76.2)	18 (66.7)	0.471	1 (33.3)	0 (0)	1.000
Coronary heart disease	8 (38.1)	11 (40.7)	0.853	1 (33.3)	0 (0)	1.000
Previous TIA/stroke	1 (4.8)	1 (3.7)	1.000	0 (0)	0 (0)	-
Previous cerebral infarction	4 (19.0)	2 (7.4)	0.383	2 (66.7)	0 (0)	0.400
Previous cerebral hemorrhage	0 (0)	1 (3.7)	1.000	0 (0)	0 (0)	-
NIHSS on admission	17 (12–25)	15 (11–19)	0.219	-	-	-
OTT, min	280 (201–348)	238 (180–340)	0.513	-	-	-
Fasting blood glucose, mmol/L	5.80 (5.22–7.71)	5.67 (5.06–6.72)	0.693	-	-	-
Platelets (×1000/μL)	154 (127–186)	172 (144–201)	0.271	-	-	-
PT/INR	1.04 (1.02–1.19)	1.01 (0.95–1.25)	0.257	-	-	-
Intravenous thrombolysis	12 (57.1)	14 (51.9)	0.715	0 (0)	0 (0)	-
Anterior circulation stroke	20 (95.2)	22 (81.5)	0.211	3 (100)	1 (50.0)	0.400
Posterior circulation stroke	1 (4.8)	5 (18.5)	0.211	0 (0)	1 (50.0)	0.400
Stroke etiology (TOAST)						
LAA						
CE						
Other						

Data are mean±SD, n (%) or median (IQR). LAA: large artery atherosclerosis. CE: cardioembolism. IQR: interquartile range. TIA: transient ischemic attack. NIHSS: National Institutes of Health Stroke Scale. OTT: onset to treatment time. PT/INR: prothrombin time and international normalized ratio.

### Treatment effects

The effects of smoking status on treatment effects are shown in Table 2. In all patients, logistic regression analysis indicated that the proportions of mild stroke (NIHSS, 0–4) at 24 hours and at 3 days were both comparable between smokers and non-smokers (21.9% vs 21.9%, p=1.000; 29.7% vs 34.4%, p=0.570; respectively). However, at discharge, smokers presented with a lower frequency of mild stroke than non-smokers (31.3% vs 57.8%, OR=0.33; p=0.003). For the LAA subtype, results revealed that the rates of mild stroke after treatment were all comparable between smokers and non-smokers (27.5% vs 22.9%, p=0.645, at 24 hours; 37.5% vs 31.4%, p=0.582, at 3 days; 40.0% vs 45.7%, p=0.618, at discharge). For the CE subtype, smokers showed lower rate of mild stroke at 24 hours (9.5% vs 22.2%) and at 3 days (14.3% vs 40.7%) than non-smokers, and the difference was not statistically significant (p>0.05), However, smokers had a lower proportion of mild stroke at discharge compared to non-smokers ( 14.3% vs 70.4%, OR=0.044; p<0.001 ), which is similar to the outcome in all patients.

**Table 2:** Effects of smoking status on treatment effects in patients with different ischemic stroke subtypes, Nanjing, China, 2014–2018 (N=128)

*Stroke scale*	*Smokers*	*Non-smokers*	*OR (95% CI)*	*p*
**All Patients**				
NIHSS_24h, 0–4	14/64 (21.9)	14/64 (21.9)	1.00 (0.43–2.31)	1.000
NIHSS_3d, 0–4	19/64 (29.7)	22/64 (34.4)	0.81 (0.38–1.70)	0.570
NIHSS at discharge, 0–4	20/64 (31.3)	37/64 (57.8)	0.33 (0.16–0.69)	0.003
**LAA**				
NIHSS_24h, 0–4	11/40 (27.5)	8/35 (22.9)	1.28 (0.45–3.66)	0.645
NIHSS_3d, 0–4	15/40 (37.5)	11/35 (31.4)	1.31 (0.50–3.41)	0.582
NIHSS at discharge, 0–4	16/40 (40.0)	16/35 (45.7)	0.79 (0.32–1.98)	0.618
**CE**				
NIHSS_24h, 0–4	2/21 (9.5)	6/27 (22.2)	0.37 (0.07–2.05)	0.254
NIHSS_3d, 0–4	3/21 (14.3)	11/27 (40.7)	0.24 (0.06–1.03)	0.054
NIHSS at discharge, 0–4	3/21 (14.3)	19/27 (70.4)	0.07 (0.02–0.31)	<0.001

Data are event numbers/total numbers (%), unless indicated otherwise. NIHSS: National Institutes of Health Stroke Scale. LAA: large artery atherosclerosis. CE: cardioembolism. CI: confidence interval.

### Intracerebral hemorrhage

The effects of smoking status on intracerebral hemorrhage are summarized in Table 3. In all patients, smokers showed higher incidences of sICH and In-hospital ICH compared to non-smokers (9.4% vs 4.7%; 28.1% vs 18.8%; respectively), while the differences were both not statistically significant (p>0.05). For the LAA subtype, regression analysis revealed that the occurrences of sICH and In-hospital ICH were both comparable between smokers and non-smokers (7.5% vs 8.6%, p=0.865; 20.0% vs 22.9%, p=0.763; respectively). For CE subtype, 2 (9.5%) patients in smoking group and none in non-smoking group developed sICH, and no significant difference was detected (p=0.998). However, smokers had higher risk of In-hospital ICH (42.9% vs 14.8%, OR=4.673; p=0.036) than non-smokers.

**Table 3:** Effects of smoking status on intracerebral hemorrhage in patients with different ischemic stroke subtypes, Nanjing, China, 2014–2018 (N=128)

*Hemorrhage*	*Smokers*	*Non-smokers*	*OR (95% CI)*	*p*
**All Patients**				
sICH	6/64 (9.4)	3/64 (4.7)	2.10 (0.50–8.81)	0.309
In-hospital ICH	18/64 (28.1)	12/64 (18.8)	1.70 (0.74–3.89)	0.213
**LAA**				
sICH	3/40 (7.5)	3/35 (8.6)	0.87 (0.16–4.59)	0.865
In-hospital ICH	8/40 (20.0)	8/35 (22.9)	0.84 (0.28–2.55)	0.763
**CE**				
sICH	2/21 (9.5)	0/27 (0)	-	0.998
In-hospital ICH	9/21 (42.9)	4/27 (14.8)	4.31 (1.10–16.96)	0.036

Data are event numbers/total numbers (%), unless indicated otherwise. sICH: symptomatic intracerebral hemorrhage. LAA: large artery atherosclerosis. CE: cardioembolism. CI: confidence interval.

### Functional outcomes

The effects of smoking status on functional outcomes at 90 days are detailed in Table 4. In all patients, the proportion of minimal symptom (mRS, 0–1) was comparable between smokers and non-smokers (28.1% vs 25.0%, p=0.689). Smokers had a lower rate of functional independence (mRS, 0–2) compared to non-smokers (32.8% vs 48.4%), whereas the difference was not statistically significant (p=0.073). For the LAA subtype, results demonstrated that the rate of minimal symptom (37.5% vs 28.6%, p=0.414), functional independence (42.5% vs 42.9%, p=0.975) were both comparable between smokers and non-smokers. But for the CE subtype, smokers showed lower rate of functional independence (14.3% vs 55.6%, OR=0.13; p=0.006) than non-smokers. The proportion of minimal symptom was also lower in smokers compared to non-smokers (9.5% vs 22.2%), whereas the difference was not statistically significant (p>0.05).

**Table 4:** Effects of smoking status on functional outcomes in patients with different ischemic stroke subtypes, Nanjing, China, 2014–2018 (N=128)

*Outcomes*	*Smokers*	*Non-smokers*	*OR (95% CI)*	*p*
**All Patient s**				
3-mo mRS, 0–1	18/64 (28.1)	16/64 (25.0)	1.17 (0.54–2.57)	0.689
3-mo mRS, 0–2	21/64 (32.8)	31/64 (48.4)	0.52 (0.25–1.06)	0.073
**LAA**				
3-mo mRS, 0–1	15/40 (37.5)	10/35 (28.6)	1.50 (0.57–3.97)	0.414
3-mo mRS, 0–2	17/40 (42.5)	15/35 (42.9)	0.99 (0.39–2.47)	0.975
**CE**				
3-mo mRS, 0–1	2/21 (9.5)	6/27 (22.2)	0.37 (0.07–2.05)	0.254
3-mo mRS, 0–2	3/21 (14.3)	15/27 (55.6)	0.13 (0.03–0.56)	0.006

Data are event numbers/total numbers (%), unless indicated otherwise. mRS: modified Rankin Scale. LAA: large artery atherosclerosis. CE: cardioembolism. CI: confidence interval.

## DISCUSSION

Many studies have investigated the relationship between smoking status and clinical outcomes in stroke patients treated with IVT with conflicting results^7,9,11,20-22^. Moulin et al.^20^ found that a favorable outcome was observed in smokers who received IVT. Aries et al.^22^ showed that smoking did not affect sICH or favorable outcomes at 3 months in AIS patients treated with IVT. Such conflicting results may be related to differences in age, ethnicity and risk factors prevalence between smokers and non-smokers. However, the relationship between smoking and clinical outcomes in Asian patients with MT is unknown. Therefore, we investigated whether smoking had an influence on outcomes in Chinese AIS patients who received MT.

Our study built on this research by focusing on the subtypes of ischemic stroke. We found that smoking has differential effects on clinical outcomes based on LAA or CE subtype. In MT-treated patients with CE subtype, smoking was associated with a decreased chance of mild stroke (NIHSS 0–4 at discharge), favorable outcome (3-month mRS 0–2), and an increased risk of In-hospital ICH, whereas there was no such association in stroke patients with the subtype of LAA. Therefore, the association between smoking and unfavorable outcomes after MT might depend on the ischemic stroke subtype.

First, in MT-treated patients, we discovered a negative association between smoking and the treatment effect (NIHSS 0–4 at discharge) in patients with CE stroke subtype (OR=0.07; 95% CI: 0.02–0.31; p<0.001). However, in IVT-treated patients, Ovbiagele et al.^21^ reported that smokers had a significantly greater improvement of their median NIHSS scores at 24 hours from the baseline than in non-smokers. Tong et al.^10^ found that smoking could be related to a better chance of functional independence if their subtype of stroke was non-cardioembolic in patients treated with IVT.

Second, in MT-treated patients, we found that CE was the underlying reason of unfavorable outcomes for the smokers, but in the LAA subgroup the odds of clinical outcomes were equally distributed between smokers and non-smokers. Moreover, Wang et al.^23^ discovered that stroke patients with CE subtype had much worse clinical outcomes at follow-up at 3 months compared with those with LAA. However, in smokers treated with IVT, Tong et al.^10^ reported that non-cardioembolic stroke is considered to be an independent factor for favorable outcomes.

Third, in this study we did not find a statistical difference in sICH between smokers and non-smokers after MT treatment. Smoking status had no impact on risk of sICH, which is in line with the literature^7,22^. Because of low event rate of sICH and small patient numbers, these results should be interpreted with caution. However, in IVT-treated patients, patients with CE have more sICH and relatively higher risk of hemorrhage^24,25^ compared with those with LAA, similar results were found by Strbian and Goldstein^26,27^. Also, Tong et al.^10^ discovered that smoking could be related to a lower risk of sICH in LAA patients treated with IVT. In addition, in MT-treated patients with CE stroke subtype, we found an adverse effect of smoking in increasing the rate of In-hospital ICH (OR=4.31; 95% CI: 1.10–16.96; p=0.036). Further research is needed to better elucidate the effect of smoking on occurrence of In-hospital ICH.

We found that CE instead of LAA was associated with unfavorable outcomes for the smokers with MT. It is generally believed that CE emboli are rich in fibrin^23^ and smokers have significantly increased plasma levels of fibrinogen compared with non-smokers, which increases the probability of thrombosis^28^. It has been estimated that up to 50% of the increased risk of cardiovascular disease in smokers may be related to increased plasma concentrations of fibrinogen^29^. The formation of fibrin-rich clots in smokers is based on the continuous inflammatory stimulation to cells evolved by nicotine, which leads to elevated levels of fibrinogen^28^.

Smoking also induces a wide variety of physiological responses, some of which appear likely to be involved in accelerating atherogenesis or increasing the possibility of thrombus formation^30^, which is associated with a hypercoagulable state mediated by increased hematocrit and fibrinogen levels, higher fibrin-rich clots, and impaired endogenous fibrinolytic ability, so arterial occlusions in smokers may be rather thrombogenic^31,32^. These effects are mediated by platelet activation, increased fibrinogen and proinflammatory cytokine^28,33^. In addition, another theory suggested that this effect is mediated by nicotine induced increase in the levels of plasminogen activator 1 secreted by the brain endothelium^32^. Nicotine increases brain endothelial cell PAI-1 mRNA expression and protein production via PK-C-dependent pathway, which explains why smoking may be associated with a predisposition to thrombosis^21^.

### Strengths and limitations

To the best of our knowledge, this is the first study to explore the impact of smoking on clinical outcomes in Chinese AIS patients after MT. A potential strength of this study is the prospective nature of the protocol, providing identical monitoring of the patients in this three-center study. Furthermore, the data are of high quality because clinical data were collected systematically and prospectively at baseline and at 3 months follow-up by certified neurologists.

Our study has some limitations. First, this study was an observational, non-randomized cohort. Second, we had no record of the quantity of smoking (e.g. pack-years), leading to high heterogeneity in our smoking cohort and hindering a differentiation of heavy versus mild smokers. Third, the sample size enrolled in this study was relatively small, which might have led to bias and affected the reliability of results. Future large-scale studies should be performed. Finally, we studied the relationship between smoking and MT only in Chinese patients. Racial differences may have an impact on the relationship observed in this analysis, so our findings should be interpreted cautiously and need to be confirmed by prospective cohort studies in different populations, and so cannot easily be extrapolated to other populations.

## CONCLUSIONS

In AIS patients undergoing MT, smoking could be related to higher risk of In-hospital ICH, and lower rates of mild stroke at discharge and functional independence if their stroke subtype is cardioembolism.
